# Source code analysis dataset

**DOI:** 10.1016/j.dib.2019.104712

**Published:** 2019-10-24

**Authors:** Ben Gelman, Banjo Obayomi, Jessica Moore, David Slater

**Affiliations:** Machine Learning Group, Two Six Labs, 901 N. Stuart St, Suite 1000, Arlington, VA, 22203, USA

**Keywords:** Source code, Code comments, Bug detection, Static analysis

## Abstract

The data in this article pair source code with three artifacts from 108,568 projects downloaded from Github that have a redistributable license and at least 10 stars. The first set of pairs connects snippets of source code in C, C++, Java, and Python with their corresponding comments, which are extracted using Doxygen. The second set of pairs connects raw C and C++ source code repositories with the build artifacts of that code, which are obtained by running the make command. The last set of pairs connects raw C and C++ source code repositories with potential code vulnerabilities, which are determined by running the Infer static analyzer. The code and comment pairs can be used for tasks such as predicting comments or creating natural language descriptions of code. The code and build artifact pairs can be used for tasks such as reverse engineering or improving intermediate representations of code from decompiled binaries. The code and static analyzer pairs can be used for tasks such as machine learning approaches to vulnerability discovery.

**Table:** Specifications Table

Subject	Computer Science (General)
Specific subject area	Analysis of source code and related artifacts, including the code's comments, build artifacts, and static analysis output.
Type of data	File system directories Binary Text
How data were acquired	Data were collected using GitHub's GraphQL API [4] in order to download projects satisfying the conditions of having a redistributable license and at least 10 stars. Pairs of source code and their comments were extracted using Doxygen. Pairs of source code and their build artifacts were extracted by running the make command. Pairs of source code and their static analysis output were extracted using the Infer static analyzer.
Data format	Raw Filtered
Parameters for data collection	The source code must have come from GitHub with a redistributable license. The licenses were manually selected to ensure the code and any derivates were redistributable. We required a minimum project rating of 10 stars in order to account for quality control.
Description of data collection	By distributing computation, we queried the GitHub GraphQL API and downloaded 108,568 projects in approximately three weeks. Doxygen ran across the corpus in four weeks. The build commands ran in two weeks. The static analyzer ran in one week. The data was stored in license-compliant directory structures.
Data source location	Institution: Machine Learning Group, Two Six Labs, 901 N. Stuart St, Suite 1000, Arlington, VA 22203, USA.
Data accessibility	Repository name: Zenodo DOI to code and comment pairs: http://doi.org/10.5281/zenodo.3472050 DOI to code and build artifacts: http://doi.org/10.5281/zenodo.3472049 DOI to code and static analysis output: http://doi.org/10.5281/zenodo.3472048

**Table:** 

**Value of the Data** • These data are useful because they concentrate months of curation on redistributable source code. This allows users of these data to redistribute modifications and derivatives of the code in their own work. • The data primarily benefit researchers that are interested in various source code analysis tasks and the ability to freely redistribute the results of their work. In particular, the data provide relationships between source code, its comments, the results of its build processes, and a static analysis of the projects. • These data can be used to research problems such as predicting comments for given code [5], reverse engineering source code from binaries and build artifacts, and predicting static analysis output given a limited view of the project's original source code.

## 1 Data

The code and comment pairs are a compilation of code blocks and their related comments. Doxygen [2] successfully ran on 106,304 (of 108,568) different GitHub [1] projects. A total of 16,115,540 code-comment pairs were obtained by running Doxygen on C, C++, Java, and Python projects. The source code in these pairs can be of various granularities (classes, methods, functions, and variables), so there are potentially many code-comment pairs per individual source code file. The total count is over each individual code-comment pair, not over the number of contributing source code files. These data provide an association between source code and a description of that code. The data directory contains one directory for each project downloaded from GitHub. These project directories are named with the GraphQL ID from GitHub's GraphQL API. In each of these GraphQL-ID labeled directories, there is a license.txt, a url.txt, and a derivatives directory. The license.txt contains the license for the original project, the url.txt contains a link to the original project on GitHub, and the derivatives directory contains the output of running Doxygen on the project. The Doxygen output is a json file, structured as a dictionary with a “contents” field, where the value of that field is a list of lists containing 3 elements each. The following is a mock example of that structure: {“contents”: [[<path1>, <snippet1>, <comment1>], [<path2>, <snippet2>, <comment2>], …]}. The “path” is a filepath relative to the original project from which the code and comment were obtained. The “snippet” is the actual body of the source code. The “comment” is the corresponding comment. For convenience, there is also an initialize.py python script that iterates through all of the json files in the data directory and stores them in an SQLite database called “all_data.db”. The license.txt and url.txt files are necessary to fulfill licensing requirements for redistribution. We used the original license filenames, so they are not always named “license.txt”, but always contain “license”, “licence”, or “copy” in the filename.

The code and build artifact pairs are a compilation of source code projects and their related build outputs. The build process, which consisted of running the make command [6], successfully ran on 3049 different GitHub projects. Over 30,000 build outputs were produced from C and C++ projects. The build outputs are the results of running a particular project's make command. These *derivatives* include executables, object files, including libraries (.o files,.so files,.a files), and other project-specific build artifacts. The output was accepted as long as the make command completed without error; thus, there is no guarantee that every project will contain every type of artifact. Furthermore, some make files perform cleanup of object files after generating the final executable; for such projects, the object files will not be available. These data provide an association between source code and the build artifacts of that code. The data directory contains one directory for each project downloaded from GitHub. These project directories are named with the GraphQL ID from GitHub's GraphQL API. In each of these GraphQL-ID labeled directories, there is a license.txt, a url.txt, source directory, and a derivatives directory. The license.txt contains the license for the original project, the url.txt contains a link to the original project on GitHub, the source directory contains the original code, and the derivatives directory contains the outputs from building the project, which include the previously mentioned files.

The code and static analysis dataset is a compilation of source code projects and their outputs from running the static analysis tool, Infer [3], on 3170 different C and C++ GitHub projects. These data provide an association between source code and a static analysis of that code. The data directory contains one directory for each project downloaded from GitHub. These project directories are named with the GraphQL ID from GitHub's GraphQL API. In each of these GraphQL-ID labeled directories, there is a license.txt, a url.txt, a source directory, and a derivatives directory. The license.txt contains the license for the original project, the url.txt contains a link to the original project on GitHub, the source directory contains the original code, and the derivatives directory contains the output of Infer.

## 2 Experimental design, materials, and methods

We designed our data collection using GitHub's GraphQL API to locate projects that satisfied our requirements. The GraphQL API allowed us to functionally encode our requirements to query the tremendous quantity of source code on GitHub. Our main concerns for the data included the ability to freely redistribute modifications or derivatives of the code and a reasonable expectation of quality for each project. To address redistribution, we manually selected 15 acceptable licenses: MIT, Apache-2.0, GPL-2.0, GPL-3.0, BSD-3-Clause, AGPL-3.0, LGPL-3.0, BSD-2-Clause, Unlicense, ISC, MPL-2.0, LGPL-2.1, CC0-1.0, EP1-1.0, and WTFPL. To address code quality, we used GitHub's starring system to set a threshold of 10 or more stars. We chose this threshold empirically, during the process of setting up our project-mining infrastructure, after viewing many repositories with a range of star values. Additionally, we accepted projects from a variety of programming languages, which GitHub enumerates in a list of popular languages, that have Doxygen plugins. By setting the license, quality, and language parameters, we were able to receive project URLs from GraphQL. The query string used is shown in the Appendix.

Using the project URLs returned from the GraphQL queries, we ran curl commands in parallel to download the master branch of each GitHub repository. We terminated the downloads after 3 weeks, resulting in approximately 8 terabytes of data. After all the downloads completed, we ran three utilities to extract data. These processes were run to completion; we did not terminate them early.

We used Doxygen to extract code-comment pairs, which ran and finished in a total of four weeks. We used Doxygen version 1.8.11. We modified the “FILE_PATTERNS” variable in the doxyfile configurations to include the following extensions:.c,.cc,.cxx,.cpp,.c++,.h,.hh,.hxx,.hpp,.h,.java, and.py. We did not make any other modifications to the default settings.

We used the make command to build the projects, which ran and finished in a total of two weeks. We did not perform any additional dependency resolution beyond what was available inside the individual source code projects. We also did not attempt to modify any compilation options or flags, as those were defined in the individual make files. The target architecture was Ubuntu 16.04.1 x86_64. We allowed the projects to run any of the four compilers: g++ 4:6.3.0–4 amd64, g++ 6.3.0–18 + deb9u1 amd64, gcc 4:6.3.0–4 amd64, and gcc 6.3.0–18 + deb9u1 amd64.

We used Infer to obtain a static analysis of the code, which ran and finished in a total of one week. We chose Infer as opposed to other static analyzers (e.g., Clang Static Analyzer) due to its recency and popularity amongst large software projects, which is due in part to its scalability. We used Infer version v0.16.0 with the command “infer run -- make”. We did not change any other parameters of the infer tool. The target architecture and potential compilers are the same as the ones used for the project building.

After the artifact generation process, we packaged the data into a legally compliant format. For every project, we created a directory that included the original project's license, a link back to the original project, and any source code that was used in the creation of the artifacts we have provided.

## Figures and Tables

**Figure:**
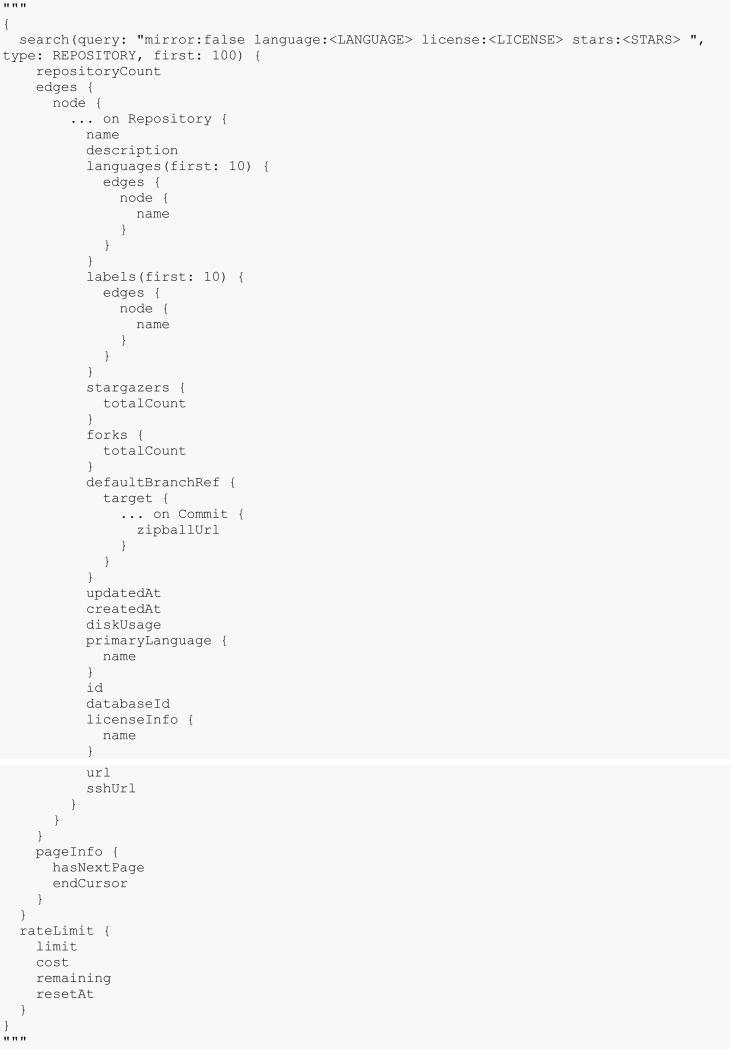


## Appendix

The following string shows the GraphQL query that we used to mine the GitHub projects of interest. We downloaded a total of 353,361 projects, which were then filtered to only include C, C++, Python, and Java projects, a total of 108,568 projects.
